# Basal activity of PINK1 and PRKN in cell models and rodent brain

**DOI:** 10.1080/15548627.2023.2286414

**Published:** 2023-12-02

**Authors:** Jens O. Watzlawik, Fabienne C. Fiesel, Gabriella Fiorino, Bernardo A. Bustillos, Zahra Baninameh, Briana N. Markham, Xu Hou, Caleb S. Hayes, Jenny M. Bredenberg, Nicholas W. Kurchaba, Dominika Fričová, Joanna Siuda, Zbigniew K. Wszolek, Sachiko Noda, Shigeto Sato, Nobutaka Hattori, Asheeta A. Prasad, Deniz Kirik, Howard S. Fox, Kelly L. Stauch, Matthew S. Goldberg, Wolfdieter Springer

**Affiliations:** a Department of Neuroscience, Mayo Clinic, Jacksonville, FL, USA; b Neuroscience PhD Program, Mayo Clinic Graduate School of Biomedical Sciences, Jacksonville, FL, USA; c Department of Neurology, Faculty of Medical Sciences in Katowice, Medical University of Silesia, Katowice, Poland; d Department of Neurology, Mayo Clinic, Jacksonville, FL, USA; e Department of Neurology, Juntendo University Graduate School of Medicine, Tokyo, Japan; f Faculty of Medicine and Health, School of Medical Sciences, University of Sydney, Sydney, NSW, Australia; g Department of Experimental Medical Science, Lund University, Lund, Sweden; h Department of Neurological Sciences, University of Nebraska Medical Center, Omaha, NE, USA; i Center for Neurodegeneration and Experimental Therapeutics, Department of Neurology, University of Alabama at Birmingham, Birmingham, AL, USA

**Keywords:** Mitophagy, parkin, Parkinson disease, PINK1, PRKN, ubiquitin

## Abstract

The ubiquitin kinase-ligase pair PINK1-PRKN recognizes and transiently labels damaged mitochondria with ubiquitin phosphorylated at Ser65 (p-S65-Ub) to mediate their selective degradation (mitophagy). Complete loss of PINK1 or PRKN function unequivocally leads to early-onset Parkinson disease, but it is debated whether impairments in mitophagy contribute to disease later in life. While the pathway has been extensively studied in cell culture upon acute and massive mitochondrial stress, basal levels of activation under endogenous conditions and especially *in vivo* in the brain remain undetermined. Using rodent samples, patient-derived cells, and isogenic neurons, we here identified age-dependent, brain region-, and cell type-specific effects and determined expression levels and extent of basal and maximal activation of PINK1 and PRKN. Our work highlights the importance of defining critical risk and therapeutically relevant levels of PINK1-PRKN signaling which will further improve diagnosis and prognosis and will lead to better stratification of patients for future clinical trials.

## Introduction

The ubiquitin (Ub) kinase PINK1 and the Ub ligase PRKN together orchestrate a critical quality control mechanism to maintain a healthy and functional cellular pool of mitochondria (reviewed in [1]). Damaged mitochondria are selectively identified and coordinately labeled by both enzymes with serine-65 phosphorylated Ub (p-S65-Ub). Such tagged mitochondria are then recognized by autophagy receptors to engulf and digest the defective organelles via the autophagy-lysosome system [2–5]. Complete loss of PINK1 or PRKN function unequivocally leads to death of dopamine (DA) neurons and early-onset Parkinson disease (PD) [6,7], but the impact of reduced expression or activity on disease risk later in life is debated. Also, both genes are broadly expressed, but the requirement for and extent of PINK1-PRKN signaling and their contribution to basal mitophagy in different cell types and especially *in vivo* remain unclear [8–10].

Analyses of PINK1-PRKN mitophagy are generally complicated by the stress-induced activation and the transient nature from an acute initiation to an effective clearance. The extent of both mitochondrial dysfunction and lysosomal degradative capacity determines mitophagy flux (reviewed in [1]). Under normal conditions, full-length PINK1 protein is barely detectable but rapidly stabilizes upon mitochondrial damage locally in the outer membrane and auto-phosphorylates. This allows PINK1 to phosphorylate preexisting Ub molecules on the mitochondrial surface that recruit and activate the auto-inhibited Ub ligase PRKN to these subdomains where it ubiquitinates a multitude of substrate proteins without apparent sequence or structural specificity [11]. Once fully active, following PINK1 phosphorylation of its Ub-like domain, the products of PRKN become additional substrates for PINK1 thereby amplifying the p-S65-Ub signal to coat damaged organelles. Upon PINK1-dependent activation, PRKN auto-ubiquitylates and is degraded alongside damaged mitochondria in a dynamic process.

While this cascade has been well established in cell culture upon acute and massive mitochondrial depolarization, much less is known about the levels of PINK1 and PRKN activation under basal (non-stress) conditions. Given the typically only very localized, low-level activation on mitochondria at baseline and the continuous flux through the autophagy-lysosome system, paired with lack of sensitive tools, it has been challenging to measure these dynamic events under non-stress conditions. We previously established an electrochemical enzyme-linked immunosorbent assay (ELISA) to robustly and reliably detect p-S65-Ub in cells, tissues, and biofluids from human and animal models [12]. With this, we found significant differences between brain p-S65-Ub levels in wild type (WT), *pink1* or *prkn* knockout (KO) mice under normal conditions suggesting they have a biochemical deficit despite the lack of overt phenotypes at younger ages [13]. However, it remained unclear if there is a low critical amount of PINK1 and PRKN that is required for sufficient pathway activation in different cell types both at baseline and upon stress.

Here, in addition to brains from mice completely lacking *Pink1* or *Prkn (*-/-), we studied samples from heterozygous (+/-) animals. We demonstrate that even reduced expression of the Ub kinase PINK1 significantly diminishes p-S65-Ub levels in mouse brain and human cells at baseline, while only complete loss of the Ub ligase PRKN has an equivalent effect size, at least under non-stress conditions. Comparing younger and older mice, we highlight age-dependent and brain region-specific effects for the activation of the PINK1-PRKN pathway, which we further confirmed in samples from corresponding rat KO models of *Pink1* or *Prkn*. Moreover, we translated our findings from rodent brain to human specimens including PD patient-derived skin fibroblasts, induced pluripotent stem cells (iPSCs), and differentiated DA neurons at baseline and under acute mitochondrial stress. Last, we establish that ablation of PINK1 leads to a robust and reproducible increase of (most likely inactive) PRKN protein, which we suggest might reflect the cell- or tissue-specific, Ub-dependent mitophagy rate at baseline. Our study not only demonstrates the basal, though low-level activation of PINK1 and PRKN, but highlights potentially critical thresholds and therapeutically relevant levels that may have implications for the development of future biomarkers and treatments.

## Results

### Loss of PINK1 modulates PRKN protein and p-S65-Ub levels in mouse brain

**Figure 1.**
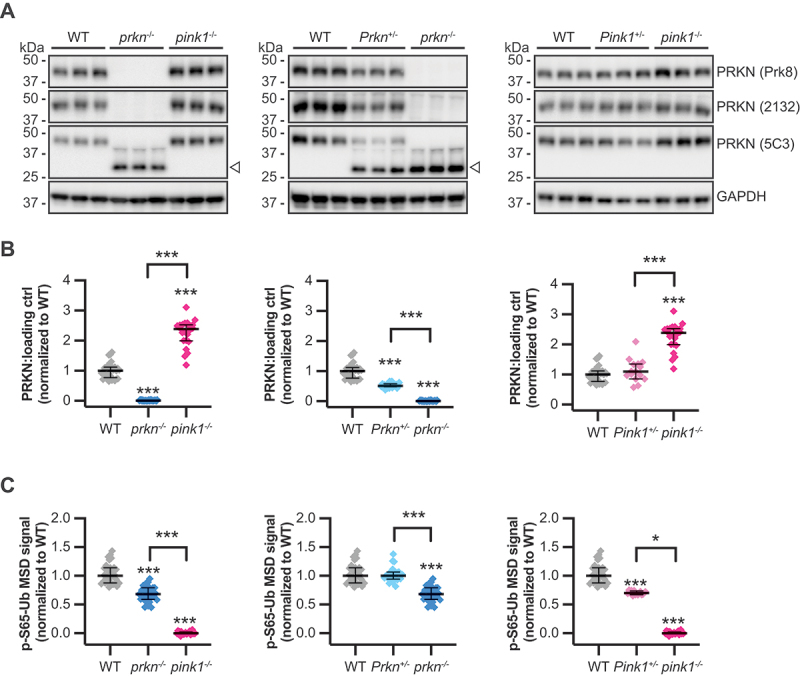
Complete loss of PINK1 in mouse brain stabilizes PRKN protein while partial loss dampens p-S65-Ub levels. PRKN and p-S65-Ub levels were determined at basal conditions in hemibrain lysates from mice with the following genotypes: WT (*n* = 29), heterozygous *Prkn*^*+/-*^ (*n* = 23), homozygous *prkn*^*-/-*^ (*n* = 29), heterozygous *Pink1*^*+/-*^ (*n* = 15), homozygous *pink1*^*-/-*^ (*n* = 22). For better comparison, mice were grouped and analyzed by genotype: WT, *prkn*^*-/-*^, *pink1*^*-/-*^ (left); WT, *Prkn*^*+/-*^, *prkn*^*-/-*^ (middle); and WT, *Pink1*^*+/-*^, *pink1*^*-/-*^ (right). (A) Representative western blots obtained with three different anti-PRKN antibodies (Prk8, 2132, and 5C3) are shown together with the loading control GAPDH. An open arrowhead labels the truncated PRKN-EGFP fusion protein produced in the *prkn*^*-/-*^ samples. (B) PRKN (Prk8) protein levels were assessed by densitometry with data points shown as ratio PRKN divided by GAPDH (median ± interquartile range [IQR]). Quantification of PRKN protein using the other two antibodies can be found in Fig. S1A and B. (C) p-S65-Ub levels were quantified by sandwich ELISA with data points shown as median ± IQR. Data was analyzed by using a Kruskal-Wallis test combined with Dunn’s multiple comparison test (***, *p* < 0.0005; *, *p* < 0.05). Comparisons to the WT are shown on top of data points, while comparisons between other genotypes are indicated by brackets.

To biochemically determine basal enzymatic activities of PINK1 or PRKN, we analyzed hemibrain lysates from WT mice and animals with partial (+/-) or complete (-/-) loss of *Pink1* or *Prkn*. We first focused on PRKN protein and obtained coherent immunoblot results with three independent antibodies (Figure 1A). All antibodies showed absence of full-length PRKN protein in brain lysates from homozygous and reduced levels from heterozygous *Prkn*^*+/-*^ mice (Figure 1B). Consistent with the design of the mutant strain, in which EGFP was inserted into exon 3 to effectively disrupt the *Prkn* gene [14], an antibody against the N terminus of PRKN (PRKN 5C3) detected a band corresponding to a truncated form of PRKN (aa 1–95) fused to EGFP. Regardless of the epitope region, all three antibodies showed a conspicuous increase of full-length PRKN protein in *pink1^−^*^*/-*^ brain lysates compared to samples from *Pink1*^*/-*^ animals or WT controls. Quantification of the immunoblot signal revealed more than 2.2-fold higher protein levels of the Ub ligase PRKN upon complete loss of the Ub kinase PINK1 compared to WT controls with the Prk8 antibody (Figure 1B). The same trend was found with additional PRKN antibodies **(Fig. S1A and B)**. We next measured levels of *Pink1* and *Prkn* mRNA in brains from WT and homozygous *pink1* (or *prkn*) KO animals but did not find a matching increase in gene expression **(Fig. S1C)**. Since levels of the truncated PRKN-EGFP fusion were not increased in a *pink1*^*-/-*^ mutant background **(Fig. S1D and E)** as would have been expected from a compensatory, transcriptional upregulation of *Prkn* gene expression, we conclude that this regulation of PRKN occurs on protein level.

In addition to PRKN protein levels, we employed a sandwich ELISA on a Meso Scale Discovery (MSD) platform to quantify amounts of p-S65-Ub, the joint product of the Ub kinase-ligase pair PINK1-PRKN [12]. Compared to WT controls, p-S65-Ub levels were reduced by ~ 35% in *prkn*^*-/-*^ brains, while the mitophagy tag was undetectable in *pink1*^*-/-*^ lysates (Figure 1C). Despite a significant reduction of PRKN protein in heterozygous *Prkn*^*+/-*^ brains, p-S65-Ub levels were indistinguishable from WT animals. However, in brains from heterozygous *Pink1*^*+/-*^ animals, p-S65-Ub levels were significantly reduced relative to WT controls, which was comparable in extent to mice with complete loss of PRKN function. Accordingly, spearman correlations between p-S65-Ub and PRKN protein levels in WT, *Prkn*^*+/-*^, and *prkn*^*-/-*^ brains were positive and highly significant (*r* = 0.72, *p* < 0.0001) **(Fig. S1F)**. Altogether these data identify critical levels of the enzymes PINK1 and PRKN and their respective contributions to the baseline levels of p-S65-Ub in mouse brain. The accumulation of PRKN protein upon complete loss of PINK1, further highlights the critical role of the Ub kinase not only for the activation of PRKN, but also the consequent turnover of the Ub ligase.

### Age- and region-specific expression of PRKN influence p-S65-Ub levels in rodent brain

For additional insights, we investigated potential influences of other biological variables on basal PINK1 or PRKN activities. Separate analysis of males and females did not reveal sex-specific changes in either brain PRKN protein or p-S65-Ub levels within any of the genotypes (**Fig. S1G**). However, we noticed an age-dependent effect. While there was no significant age difference between or amongst most genotypes, some of the *prkn*^*-/-*^ animals used in this study were older than their WT or other mutant counterparts **(Fig. S1H)**. Within the group of WT animals, which varied in age only from 2.1 to 8.5 months, no age correlation was seen for brain p-S65-Ub levels. Yet, and despite an overall dampened p-S65-Ub signal, a significant positive correlation with age was detectable in brains from *prkn*^*-/-*^ mice that ranged in age from 4.4 up to 13.9 months **(Fig. S1I)**.

To determine possible regional differences, we expanded our investigation and collected six distinct brain subregions from an independent mouse cohort of young (4.5 months) or old (24 months) WT and *prkn*^*-/-*^ animals. Comparison of PRKN protein levels between young and old WT mice revealed only minor differences between the age groups as well as across the individual brain regions (**Fig. S2A)**. However, levels of p-S65-Ub were significantly increased in most brain regions (5 out of 6) obtained from old compared to young WT animals. No such change was found in any of the respective brain regions from *prkn*^*-/-*^ mice **(Fig. S2B)**. Comparison between the two genotypes revealed significant differences in p-S65-Ub only in the cortex and the striatum of young animals. At more advanced age, levels of p-S65-Ub were found significantly reduced in 5 out of 6 brain regions from *prkn*^*-/-*^ animals compared to WT controls. Consistently, PRKN expression significantly correlated with p-S65-Ub levels across all brain regions from young and old animals **(Fig. S2C)**.

Lastly and to confirm our findings in a different species, we measured PRKN and p-S65-Ub levels in rat cerebellum and hippocampus from 8 months old WT controls and animals with complete loss of *Pink1* or *Prkn*. Consistent with the data from mice, PRKN protein levels were significantly elevated in studied brain regions from *pink1*^*-/-*^ rats (**Fig. S2D and E)**, while p-S65-Ub levels were dampened in *prkn*^*-/-*^ animals relative to WT controls at least in the hippocampus (**Fig. S2F**). It is noteworthy that in both WT and *pink1*^*-/-*^ rats, PRKN protein levels were on average 1.4-fold higher in hippocampus than cerebellum. Likewise, p-S65-Ub levels were 2.4-fold higher in hippocampus compared to cerebellum in WT animals. No significant difference in p-S65-Ub levels was observed between these two regions in *prkn*^*-/-*^ or *pink1*^*-/-*^ rat brains. Together this further supports the idea that age, brain subregion, and possibly also cell type specific differences in p-S65-Ub levels are, at least in part, driven by changes in gene expression of the Ub ligase PRKN in both mice and rats.

### Acute mitochondrial stress has opposing effects on PINK1 and PRKN protein levels

**Figure 2.**
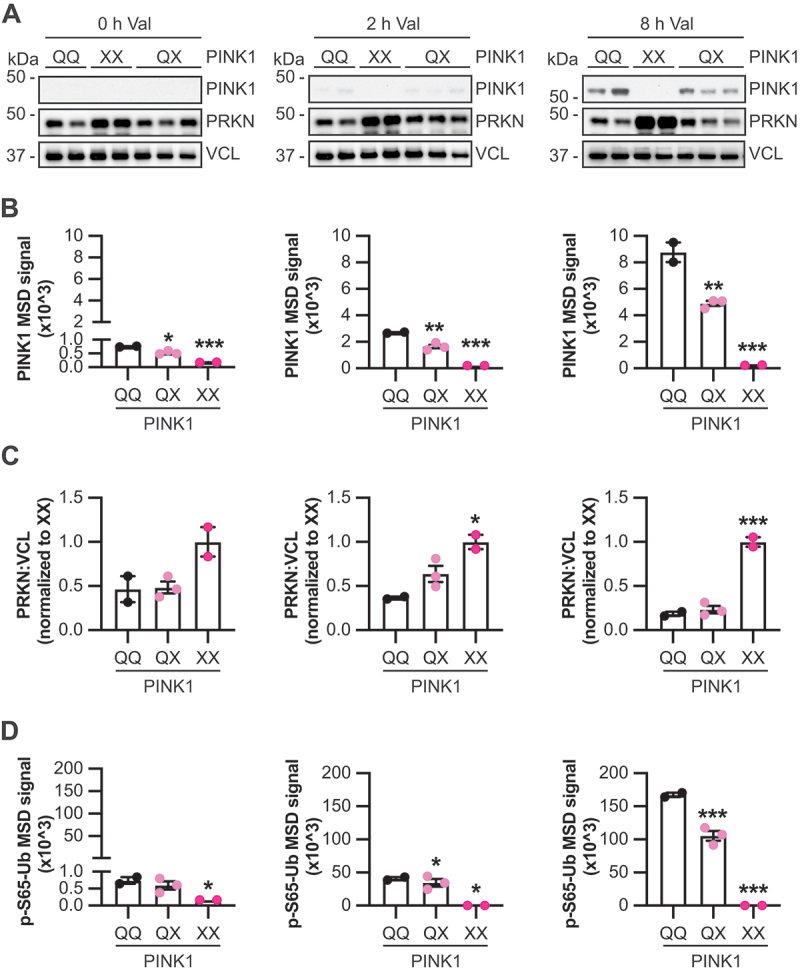
PINK1 gene dosage affects p-S65-Ub and PRKN levels in PD patients’ cells. Primary skin fibroblasts from related individuals without (QQ; *n* = 2), with one (QX; *n* = 3) or with two (XX; *n* = 2) mutant PINK1 Q456X alleles were either left untreated (0 h, left) or were treated for 2 h (middle) or 8 h (right) with 1 µM valinomycin (Val). Data is arranged by treatment groups (i.e., time points). (A) Cell lysates were analyzed by western blot using antibodies against PINK1, PRKN, and the loading control VCL (vinculin). (B) Protein levels of PINK1 were quantified by sandwich ELISA. (C) PRKN protein levels were derived by densitometry of the western blots and data points shown as a ratio of PRKN divided by VCL and normalized to PINK1^XX^. (D) p-S65-Ub levels were also quantified by sandwich ELISA. Data is shown as the mean ± standard error of the mean of biological replicates, grouped by allele count of the Q456X mutation, and analyzed by one-way ANOVA with Tukey’s multiple comparison test (***, *p* < 0.0005; **, *p* < 0.005; *, *p* < 0.05). For untreated the mean of 4 technical repeats per biological sample is shown. Asterisks on top of data points indicate individual comparison to WT controls without PINK1 mutation.

To ascertain critical levels of PINK1 and PRKN in human cells, we first analyzed primary skin fibroblasts from a family carrying the PINK1^Q456X^ mutation [15]. We confirmed the significant loss of *PINK1* mRNA caused by introduction of the premature termination codon [16], while *PRKN* mRNA levels were comparable between WT and homozygous PINK1 mutant cells (**Fig. S3A**). For biochemical analysis of PINK1, PRKN, and p-S65-Ub, fibroblasts were either left untreated (baseline) or were treated with valinomycin to acutely depolarize mitochondria and activate the pathway (Figure 2A). In the absence of stress, PINK1 protein was barely detectable by western blot. However, using an MSD ELISA for PINK1, we found that compared to cells with WT alleles (PINK1^QQ^), cells with one Q456X allele (PINK1^QX^) had significantly less PINK1 signal, and this signal was further decreased in cells with two mutant alleles (PINK1^XX^) (Figure 2B). Similar to rodent brain, we observed elevated PRKN protein levels in fibroblasts with two, but not one dysfunctional copy of the *PINK1* gene (Figure 2C). Only cells with two mutant PINK1 alleles showed lower p-S65-Ub compared to WT at baseline (Figure 2D).

Upon treatment with valinomycin (Figure 2A and B), PINK1 protein levels steadily increased in WT cells and in cells with one mutant allele but stayed undetectable in cells with two mutant alleles (**Fig. S3B**). At each time point, cells with one mutant allele had only about 50% PINK1 protein levels compared to WT. With mitochondrial stress, PRKN levels remained unchanged in cells with two mutant alleles [17], but after 2 h of valinomycin, levels of PRKN decreased in the WT cells. After 8 h valinomycin treatment, samples with one or zero mutant alleles both showed reduced PRKN levels (Figure 2C). This resulted in more significant differences in PRKN protein between WT cells and cells with two Q456X alleles from about 2.1-fold at baseline (0 h) to 5.4-fold after 8-h treatment with valinomycin. Over the same time course, p-S65-Ub levels increased almost 160-fold in WT cells (Figure 2D and S3C), while levels remained significantly lower in cells with one mutant allele, increasing only up to 110-fold. Further, cells with the PINK1^XX^ genotype did not show any p-S65-Ub increase over time.

To validate our findings in an independent, disease-relevant cell model, we turned to ReN cell VM, an immortalized neural precursor line that can be terminally differentiated into neurons [18,19]. Using CRISPR-Cas9 we created a matching set of neuronal progenitors with deletion of only one (+/-) or both alleles (-/-) of the *PINK1* gene. We first confirmed the dose-dependent reduction of *PINK1* mRNA while levels of *PRKN* mRNA remained unchanged (**Fig. S3D**). Differentiated neurons were then left untreated or were stressed for 2 or 8 h. Because of considerably lower toxicity in this neuronal model, we used the mitochondrial depolarizer carbonyl cyanide m-chlorophenylhydrazone (CCCP) as opposed to valinomycin (**Fig. S3E**). Both compounds effectively depolarize mitochondrial membranes and acutely induce PINK1 activation. As expected, PINK1 protein was barely detectable at baseline in the parental WT neurons but were reduced in PINK1^+/-^ and absent in neurons of the PINK1^−/−^ line (**Fig. S3F**). With CCCP treatment, PINK1 signal increased considerably in both WT and heterozygous KO neurons but always remained reduced in the latter. In accordance with findings from patients’ cells, neurons with complete loss of PINK1 showed significantly, about 1.7-fold, elevated PRKN protein levels even at basal conditions. Upon acute mitochondrial stress, PRKN signal strongly declined only in WT and heterozygous PINK1 mutant cells, resulting in an 8.6-fold difference between WT and PINK1^−/−^ neurons after 8 h CCCP treatment (**Fig. S3F**). Consistently, p-S65-Ub was barely detectable at baseline, but levels increased considerably with CCCP treatment in both WT and to a lesser extent in PINK1^+/-^ neurons while remaining undetectable in the corresponding PINK1^−/−^ line. Likewise, MFN2, which we monitored as a representative PRKN substrate, was ubiquitinated in PINK1^+/+^ but not in PINK1^−/−^ cells. MFN2 ubiquitination was further significantly impeded by partial loss of PINK1, increasing over the same time only 2.6-fold compared to 5.6-fold in isogenic WT neurons.

Taken together we demonstrate that while complete loss of PINK1 abolishes p-S65-Ub signaling and thereby stabilizes (inactive) PRKN protein, even partial loss of the Ub kinase (~50%) significantly dampens and delays the mitophagy response at basal conditions but especially during acute mitochondrial stress.

### Cell type and expression level of PINK1 and PRKN dictate p-S65-Ub levels

**Figure 3.**
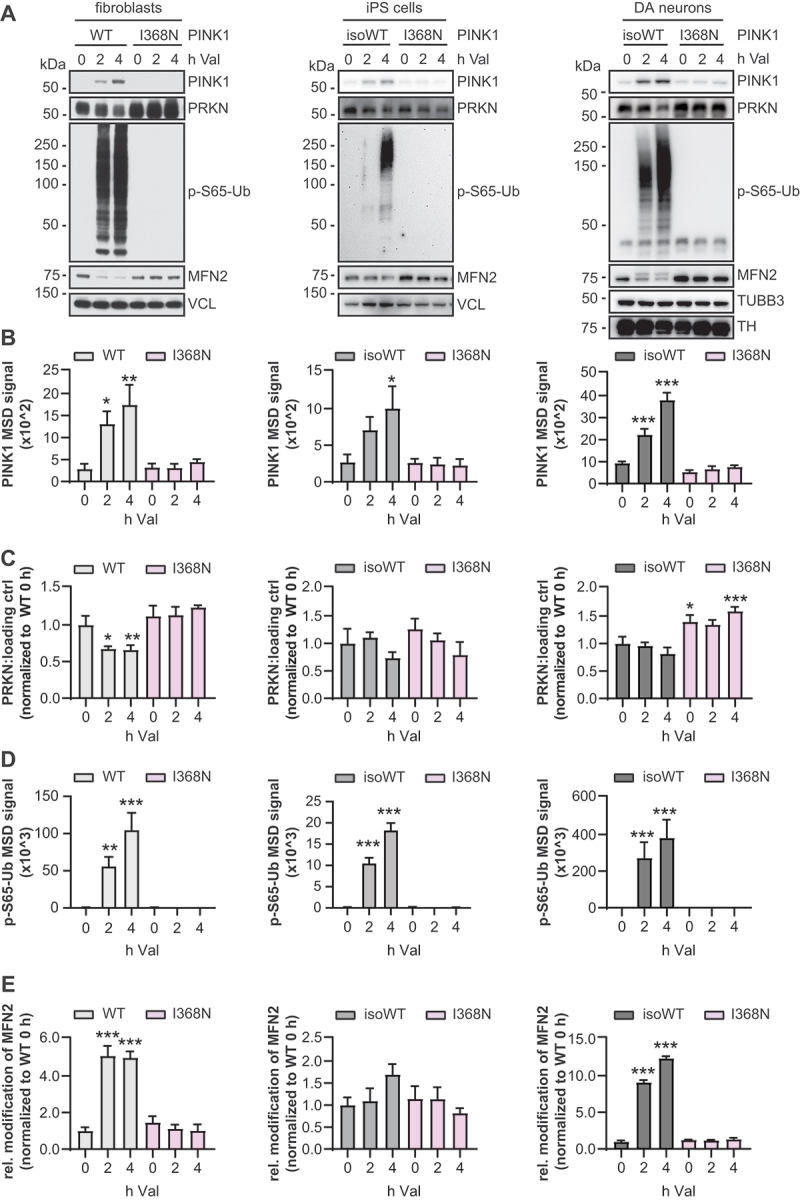
Cell type-specific expression of PINK1 and PRKN in fibroblasts, iPSCs, and midbrain DA neurons drive p-S65-Ub levels. Primary skin fibroblasts from either a control or patient with PINK1^I368N^ mutation, and undifferentiated iPS cells that were generated from the same PINK1^I368N^ patient cells and their gene-corrected counterparts (isogenic WT), as well as DA neurons generated from the same iPSC set were treated with 1 µM valinomycin (Val) for the indicated times and harvested. (A) Representative western blots show levels of PINK1, PRKN, p-S65-Ub and MFN2 for all three cell lines. VCL (vinculin) and DA neuronal markers, TUBB3/βIII-tubulin and TH (tyrosine hydroxylase), were used as loading controls. (B) Quantification of PINK1 protein levels by sandwich ELISA. (C) Densitometric analysis of PRKN western blots shown under (A) and data points displayed as PRKN divided by VCL or PRKN divided by DA neuronal markers. (D) Quantification of p-S65-Ub levels measured by sandwich ELISA. (E) Densitometric analysis of MFN2. Relative modification of MFN2 was calculated as the ratio of upper (ubiquitinated) to lower (unmodified) MFN2 band. The mean of three independent experiments for each cell type ± SD is shown. Statistical analysis was performed by one-way ANOVA followed by Bonferroni correction. (***, *p* < 0.0005; **, *p* < 0.005; *, *p* < 0.05). Asterisks on top of data points indicate individual comparison to respective WT controls without PINK1 mutation.

To expand our analyses to different cell types, we analyzed fibroblasts, induced pluripotent stem cells (iPSCs), and thereof differentiated midbrain DA neurons all from the same individual carrying a homozygous PINK1^I368N^ mutation. For comparison we used WT control fibroblasts or an isogenic iPSC line in which the I368N mutation had been corrected by CRISPR-Cas9 (isoWT). In line with our previous report that the mutation affects stabilization and Ub kinase activity [20], PINK1 protein levels were strongly reduced in the mutant cells and p-S65-Ub levels were undetectable (Figure 3A). During a short time course of up to 4 h valinomycin treatment, PINK1 increased comparably in fibroblasts (3.5-fold), iPSCs (2.4-fold), and DA neurons (3.3-fold) from the respective basal levels in the corresponding (iso)control WT cells (Figure 3B). At the same time PRKN protein levels similarly decreased upon PINK1-dependent activation by about 30% in each of the WT cell types (Figure 3C). In the absence of PINK1, PRKN protein levels were slightly elevated at baseline (1.2- to 1.5-fold) and more noticeably after 4 h depolarization (1.8- to 2.6-fold) compared to the respective (iso)controls. At the same time in the presence of PINK1, p-S65-Ub levels increased 115-fold in fibroblasts, 92-fold in iPSCs, and more than a 1000-fold in DA neurons, compared to the respective basal levels (Figure 3D). MFN2 modification always followed a similar trend to the induction of p-S65-Ub in the respective cells increasing 5.0-fold in fibroblasts, 1.7-fold in iPSCs, and more than 10-fold in DA neurons (Figure 3E).

We next compared the signals of PINK1, PRKN, p-S65-Ub, and MFN2 between the different cell types both at baseline and upon acute stress **(Fig. S3G and H)**. Basal levels of PINK1 protein were similar between fibroblasts and iPSCs but were about 2-fold higher in DA neurons. Upon valinomycin treatment and compared to iPSCs, PINK1 levels were 2-fold higher in fibroblast and 4-fold higher in DA neurons. PRKN protein levels were distinct between all three cell types at baseline, with about seven times greater levels in fibroblasts and almost 40 times more PRKN protein in DA neurons compared to iPSCs. PINK1-dependent activation caused a similar relative decline in PRKN protein levels and after 4 hours of mitochondrial depolarization, fibroblasts still contained 3.8 times and DA neurons almost 23 times the amounts of PRKN protein found in iPSCs. Similar to PINK1, baseline p-S65-Ub levels were generally very low across all cell types. Upon valinomycin treatment, p-S65-Ub swiftly and considerably increased across all cell types but rose most dramatically in DA neurons reaching almost 4.6 times the levels in fibroblasts and more than 26 times the levels seen in iPSCs. Activated PRKN ubiquitinates MFN2, with the ratio of modified (ubiquitinated) to unmodified MFN2 serving as an additional readout of PINK1-PRKN pathway activation. Following a similar trend to PINK1 and p-S65-Ub, levels of modified MFN2 were very low at baseline across cell types. Upon valinomycin treatment, levels of modified MFN2 substantially increased across cell types, with DA neurons reaching over 2.7 times the level in fibroblasts and about 8 times the level seen in iPSCs. Altogether our findings reveal differences in expression levels and activation of PINK1 and PRKN that likely meet the typical demand of the respective cell types. While PINK1 levels are relatively comparable, the extent of the mitophagy response (levels of p-S65-Ub and MFN2 modification) seem to considerably depend on PRKN protein levels that are much greater in DA neurons compared to other cell types studied herein.

## Discussion

PINK1-PRKN mitophagy has been extensively studied in cell culture upon complete mitochondrial depolarization, but basal activities in different cell types under endogenous conditions and *in vivo* have remained uncertain so far. Such studies have been hampered by the overall, only very low activation under normal conditions and the generally dynamic nature of the pathway. We here provide the outcome of comprehensive measures of PINK1, PRKN, and their joint product p-S65-Ub in cells and brain tissue. We used a sensitive ELISA to measure levels of the transient mitophagy tag p-S65-Ub that can be used as readout of both PINK1 kinase and PRKN ligase functions. With this we determined their enzymatic activities in mouse and rat brains, across individual subregions, and during aging. In addition, we established critical low basal levels and maximal activation of PINK1 and PRKN under acute mitochondrial stress in a series of patient-derived fibroblasts, gene-edited isogenic iPSCs, and DA neurons. While our study may be somewhat limited by lack of sensitive tools especially for detection of mouse PINK1 protein and with regard to spatial resolution, our novel findings may help determine critical risk and therapeutically relevant levels of PINK1 and PRKN.

Complete loss of PINK1 or PRKN causes early-onset PD, but the contribution of heterozygous mutations or reduced enzymatic activities to disease later in life is unclear. Large-scale genetic studies continuously fail to support the prevalence of haploinsufficiency on the population level [21–24]. However, it is important to note that stress, expression level, or enzymatic activity is typically not considered when determining disease risk, and certain variants may confer larger effect sizes than others [19,25]. Here we demonstrate that even partial loss of the Ub kinase PINK1 (~50% reduction) significantly reduces p-S65-Ub levels under normal conditions *in vivo* in mouse brain. Similarly, baseline p-S65-Ub levels are detectably lower upon monoallelic loss of PINK1 in various human cells including patients’ fibroblasts and gene-edited, isogenic neurons. Upon mitochondrial stress, the reduced levels of PINK1 significantly dampened and delayed the acute mitophagy response. In contrast to PINK1, partial loss of the Ub ligase PRKN had no measurable effect on p-S65-Ub in mouse brain, at least not under basal conditions. Only complete loss of PRKN showed an effect size similar in extent to partial loss of PINK1 with respect to brain p-S65-Ub levels (~35% reduction). Yet, given the current inability to reliably measure mouse PINK1 protein, we cannot exclude the influence of a proposed feedback mechanism of PRKN activation on PINK1 stabilization [11,26,27]. Regardless, our data suggest that PRKN may play an outsized role especially in DA neurons and in brain during aging. As such the effect of partial loss of PRKN needs to be further studied under disease-relevant stress and should include direct readouts of its Ub ligase activity.

While it is known that PINK1 is critically needed to activate PRKN upon pharmacological depolarization of mitochondria, we here show that this occurs already under basal conditions, in the absence of exogenous stress, albeit at much lower levels. We demonstrate that PRKN steady-state protein levels are approximately 2-fold increased in the absence of PINK1 across cell types and species analyzed herein. Although this phenomenon has been reported before, it has been attributed to a compensatory upregulation of PRKN expression in response to loss of PINK1 [28,29]. In contrast, we propose that PRKN protein accumulates in its inactive, auto-inhibited form due to the lack of activation. In further support of this notion, PRKN is rapidly activated upon acute stress, is modified and moves into larger protein complexes before its protein levels decline as it may be degraded alongside damaged mitochondria [30–32]. It is thus tempting to speculate that the accumulation of PRKN in the absence of PINK1 reflects the otherwise undetectable, basal mitophagy rates. It remains unclear though how much PINK1 and p-S65-Ub are required to effectively activate PRKN as the accumulation of PRKN was not observed in cells and tissue with only partial reduction of PINK1 (~50%) despite a significant reduction in p-S65-Ub levels (~35%). Regardless, these findings may have important implications for therapeutic approaches aiming to de-repress or activate PRKN as it may be rapidly turned over once its enzymatic function is “unleashed”, similar to other E3 Ub ligases that auto-ubiquitylate and induce their own degradation [33].

In contrast to the maximal activation seen in cultured cells upon complete depolarization, it is important to note that the herein described baseline PINK1-PRKN signaling likely occurs only very localized on individual subdomains of mitochondria. As such we may have to adjust our expectations from the *in vitro* paradigm, as absence of PINK1 or PRKN might not lead to obvious morphological abnormalities, significant changes in metabolites or mitochondrial protein content, especially not on the tissue level [34]. Moreover, mice seem to generally experience less stress during their lifespan or can better compensate with other mitophagy pathways that act in parallel and may add to the challenge in detecting activity of PINK1 and PRKN *in vivo* [9,10,35,8]. This is further in line with the lack of obvious defects in *pink1* or *prkn* KO mice under normal conditions, while more discernable phenotypes emerge with advanced age or are triggered by stress [13,36]. In addition, both age and protein aggregation independently increase p-S65-Ub levels in both mouse and human brain [12,17,37,38]. However, our data also suggest that the age-related increase in p-S65-Ub is, at least in part, dependent on expression and activity of the Ub ligase PRKN. Likewise, differences observed between individual brain subregions likely depend on their respective cell type composition and more specifically their relative expression and activity of PINK1 and PRKN [39].

When comparing human fibroblasts, iPSCs, and DA neurons cultured from the same individual, we found PINK1 to be expressed at relative comparable levels and to be stabilized similarly in response to acute mitochondrial depolarization in the different cell types. However, PRKN expression varied more considerably, and protein levels were especially high in DA neurons compared to both iPSCs and fibroblasts. Given the resulting boost in p-S65-Ub levels, and in line with earlier studies, this supports the general idea of PRKN playing a particularly important role in neurons [11,19,40]. Although this could be directly tested by modeling reduced PRKN activity in combination with disease relevant stress in cultured DA neurons compared to other brain cell types, the situation in human brain is likely much more complicated. A plethora of posttranslational modifications have been identified on PRKN from autopsy brain and it has been shown that the Ub ligase becomes insoluble with age especially in the substantia nigra [38,41–44]. However, the relative enzymatic activity of un-/modified or in-/soluble PRKN remains unclear. As such, our study further emphasizes the need to individually determine PINK1 and PRKN levels and activities both at basal conditions and upon the respective disease relevant stress.

In summary, we show that PINK1-PRKN signaling is constitutively active even at basal (non-stress) conditions both in cultured human cells and *in vivo* in rodent brain, although at extremely low levels that requires ultrasensitive assays for detection. Although some principles of the PINK1-PRKN pathway are certainly generalizable, certain aspects seem to differ considerably between species, tissues, and cell types. Both PINK1 and PRKN are broadly expressed in the brain and beyond, but their actual protein levels and enzymatic activities vary and are probably adapted to meet the specific demand of the respective cells. While complete loss of either gene function in humans invariably leads to disease, our findings highlight the urgent need to determine the remaining expression levels and enzymatic activities of PINK1 and PRKN especially for heterozygous mutation carrier that are at risk for developing PD. Effective flux through the pathway not only depends on the activation by stress and damage, but also on an adequate turnover capacity of the autophagic-lysosomal system. As such a more refined analysis considering enzymatic and biological activity should help determine risk and improve prognosis and stratification of patients for the development and testing of future biomarkers and mechanism-based therapeutics.

## Material and methods

### Ethics approval

All procedures involving animals were in accordance with the ethical standards established by Mayo Clinic and approved by the Institutional Animal Care and Use Committees at Mayo Clinic (IACUC number: A00004649), Juntendo University (IACUC number: 2020001), and at University of Alabama at Birmingham (IACUC number: 22479).

### Animal brain tissue

A total of 118 mouse hemibrains were received from Dr. Matthew Goldberg (University of Alabama at Birmingham, USA) and included 29 WT mice (15 female, 14 male), 29 homozygous *prkn*^−/−^ mice (13 female, 16 male), 23 heterozygous *Prkn*^+/-^ mice (10 female, 13 male), 22 homozygous *pink1*^−/−^ mice (10 female, 12 male) and 15 heterozygous *Pink1*^*+*/-^ mice (7 female, 8 male). Average mouse age per genotype (combined sexes) was (mean ± standard deviation [SD]): WT: 4.4 ± 1.8 month; *prkn*^−/-^: 7.7 ± 3.3 month; *Prkn*^+/-^: 5.4 ± 1.7 month; *pink1*^−/−^: 4.4 ± 0.8 month; *Pink1*^+/-^: 4.2 ± 0.1 month. Mice were anesthetized and intracardial perfused with cold phosphate-buffered saline (PBS, Invitrogen 14190235) (4°C) and brains were removed and immediately flash-frozen in liquid nitrogen.

We further received a total of 18 mouse brains dissected into six subregions each from Dr. Nobutaka Hattori’s group (Juntendo University Graduate School of Medicine, Tokyo, Japan) consisting of ~ 2-year-old male WT (*n* = 4) and *prkn*^−/−^ (*n* = 4) mice and 4.5-months-old male WT (*n* = 5) and *prkn*^−/−^ (*n* = 5) mice. Mice were generated under a C57BL/6 background [36]. Animals were anesthetized, perfused, and brain was immediately dissected into subregions and flash-frozen in liquid nitrogen as described above.

Hippocampal and cerebellar tissue from 8-month-old male Long-Evans rats with homozygous loss of *pink1* (*n* = 3) and *prkn* (*n* = 3) compared to WT (*n* = 2) were received from Charles River Laboratories (Charles River protocol number 784002B; Horizon model IDs: TGRL4760 for *prkn*^*-/-*^; TGRL4690 for *pink1*^−/−^) via the Michael J. Fox Foundation (MJFF; https://www.michaeljfox.org/research-tools-catalog, study number WIL-784002Y −04Oct2017).

### Tissue homogenization and lysis

Frozen brains were stored at −80°C and kept on dry ice and completely frozen during handling including weighing. Throughout all subsequent handling, samples were kept at 4ºC. Mouse brain tissue was homogenized in 2 ml glass homogenizer (Fisher Scientific, K885300–0002) placed on ice using 5 volumes of ice-cold Tris-buffered saline (TBS; 50 mM Tris [Millipore, G48311], 150 mM NaCl [Fisher Scientific, BP358], pH 7.4) supplemented with protease (Roche Applied Science, 11697498001) and phosphatase inhibitors (Roche Applied Science, 04906837001) relative to brain weight e.g. 500 µl of buffer for 100 mg brain tissue. Crude homogenates were aliquoted in 100 µl aliquots and flash frozen in liquid nitrogen. Tissue lysis was completed by adding 25 µl of 5× RIPA buffer (50 mM Tris, pH 8.0, 150 mM NaCl, 1% NP-40 [USB, 19628], 0.5% deoxycholate [Sigma-Aldrich, D6750], 0.1% SDS [Bio-Rad, 1610302]) to 100 µl of mouse brain homogenate on ice, and lysates were pulse vortexed for 15 seconds and incubated at 4°C for 60 min. Insoluble material, lipids, and nucleic acids were removed by serial centrifugation (2×) at 20,000 × g, 4°C for 10 min.

### Cell culture and neuron differentiation

Patient fibroblasts were cultured in DMEM (Thermo Fisher Scientific, 11965118) supplemented with 10% fetal bovine serum (FBS; Neuromics, FBS001800112), non-essential amino acids (Thermo Fisher Scientific, 11140050) and penicillin-streptomycin (Thermo Fisher Scientific, 15140122). ReN cell VM (Millipore, SCC008) were cultured on Matrigel (Corning, 354230)-coated dishes in DMEM/F12 (Thermo Fisher Scientific, 10565042) containing B27 (Thermo Fisher Scientific, 17504044), 5 U/ml heparin (Sigma Aldrich, H3149), and 50 µg/ml gentamicin (Thermo Fisher Scientific, 15750060) in the presence of 20 µg/ml EGF (epidermal growth factor; Peprotech, AF-100-15) and FGF (fibroblast growth factor; Peprotech, 100-18B). Differentiation of parental and PINK1 KO ReN cell VM was performed by withdrawal of growth factors in the presence of 2 ng/ml GDNF (Peprotech, 450–10) and 1 mM cAMP (Invivochem, V1846) for 15 days.

iPSCs with PINK1^I368N^ and gene-corrected isogenic controls (isoWT) were obtained from the NINDS Human Cell and Data Repository (NHCDR, ND50100, ND50012). The cells were tested for normal karyotype and pluripotency, and negative for mycoplasma contamination. Undifferentiated iPSCs were maintained on a Matrigel matrix (Corning, 354277) coated surface diluted in DMEM/F12 (Gibco, 10565018) and fed with mTeSR Plus (Stemcell technologies, 100–0276). Full media changes were performed every other day or when the media color changed to yellow. Cells were treated at 90% confluency. iPSCs were differentiated into DA neurons using a previously published protocol [45] with only minor modifications. In brief, once the cells reached 95% confluency, they were dissociated using Accutase (Millipore, SCR005) and plated in mTeSR Plus with 10 µM Y-27632 ROCK inhibitor (Selleckchem, S1049) by combining 1.5 wells into 1 well of a Matrigel-coated 6-well plate. After 8 h, a full-media change was performed without ROCK inhibitor, and the cells were then allowed to recover for about 16 h. Full media changes were only performed when switching from one media type to another for the first 2 weeks of differentiation, and half media changes were subsequently performed 3 times per week until harvesting at day 35. At day 21, the cells were dissociated using Accutase and seeded onto a 0.1 mg/ml poly-L-ornithine (PLO, Sigma, P3655) and 10 µg/ml laminin (Sigma, L2020)-coated 6-well plate at a 1:1 ratio. At day 25, the cells were again dissociated and seeded at 3 × 10^6 cells/well of a 6-well plate. Cells were treated with 1 µM valinomycin (Cayman Chemical, 10009152) or 20 µM CCCP (Sigma Aldrich, C2759) to induce mitochondrial depolarization.

### Genome-editing with CRISPR-Cas9

*PINK1* KO cells were generated by CRISPR-Cas9 using a guideRNA targeting the start codon of *PINK1* (CGCCTGTCGCACCGCCATGG) and as described recently [46]. Successful targeting was confirmed by Sanger sequencing of single clones. For the heterozygous *PINK1* clones, PCR amplicons were further ligated into a cloning vector and both alleles were sequenced individually to confirm heterozygosity. In addition, the cell clones were further seeded again by limited dilution and 20 subclones were Sanger sequenced to exclude that the parental colony was a mixture of a homozygous KO and WT cells. RNA and protein expression analysis was used to confirm the loss of PINK1 in differentiated ReN cells.

### Western blot and antibodies

Cells were washed twice with ice-cold PBS and harvested in RIPA buffer. Protein concentration was determined by bicinchoninic acid (Thermo Fisher Scientific, 23225). 15–20 µg of cell lysate or 30 µg of mouse tissue lysate were loaded on to 8–16% Tris-Glycine gels (Thermo Fisher Scientific, EC60485BOX) and transferred onto polyvinylidene fluoride membrane (PVDF) membranes (Millipore, IEVH00005). Membranes were blocked with 5% skim milk in TBS with 0.1% Tween (TBST) and incubated overnight with the following primary antibodies: rabbit anti-PINK1 (Cell Signaling Technology, 6946; 1:2000), mouse anti-PINK1 (Biolegend, 846201; 1:1000), mouse anti-PRKN (Millipore, MAB5521; Prk8; 1:1000–5000 for cell lysates), mouse anti-PRKN (Cell Signaling Technology, 4211; Prk8; 1:25,000 for rodent lysates]), mouse anti-PRKN (Biolegend, 865602; 5C3; 1:2,000 for rodent lysates), rabbit anti-PRKN (Cell Signaling Technology, 2132; 1:2,000 for rodent lysates), rabbit anti-p-S65-Ub (Cell Signaling Technology, 62802; 1:3000–20,000), rabbit anti-GFP (Takara/Clontech, 632460; 1:1,000 for rodent lysates) mouse anti-MFN2 (Abcam, ab56889; 1:2000), rabbit anti-TUBB3/betaIII-tubulin (Cell Signaling Technology, 5568; 1:10,000), rabbit anti-TH/tyrosine hydroxylase (Millipore, ab152, 1:1000), mouse anti-GAPDH (Meridian Life science, H86504M: 1:400,000), mouse anti-VCL/vinculin (Sigma-Aldrich, V9131; 1:500,000–1,500,000). Secondary antibodies from Jackson Immunoresearch (donkey anti-mouse IgG [715-035-150], donkey anti-rabbit IgG [711-035-152], goat anti-mouse IgG1 [115-035-205], goat anti-mouse IgG2a [115-035-206], and goat anti-mouse IgG2b [115-035-207]) were incubated for 1 h at room temperature and signal developed using Immobilon Western Chemiluminescent HRP Substrate (Millipore, WBKLS0500). Images were taken with a Chemidoc MP imaging system (Bio-Rad, Hercules, CA, USA).

### MSD ELISA

Meso Scale Discovery (MSD) ELISA against p-S65-Ub was performed as described previously [12]. MSD ELISA against PINK1 was performed using the same protocol and is described elsewhere in more detail (Baninameh *et al*., in preparation). Briefly, 96-well plates (Meso Scale Diagnostics, L15XA–3) were coated with capture antibody in 200 mM sodium carbonate buffer pH 9.7 overnight. The next day, wells were blocked with blocking buffer (1% BSA [Boston BioProducts, Inc.; *P*-753] in TBST) and lysates were added for 1 h at room temperature. After three wash steps with wash buffer (TBST) detecting antibody was added for 2 h at room temperature, followed by sulfo-tag labeled secondary antibody (Meso Scale Diagnostics, R32AC–1). Signal was measured upon adding MSD Gold Read buffer (Meso Scale Diagnostics, R92TG–2) on a MESO QuickPlex SQ120 (Meso Scale Diagnostics, Rockville, MD, USA). Results were validated using lysates from cells with absent (*PINK1* KO) or very low PINK1 expression (PINK1 Q456X fibroblasts, this study).

### RNA analysis

Total RNA was extracted using a RNeasy spin mini kit (Qiagen, 74104). Fifty ng of RNA was used for a one-step quantitative reverse transcription PCR following the manufacturer’s protocol (Bio-Rad, 1725151). Samples were run on a 384-well block on a LightCycler480 instrument (Roche Diagnostics, Rotkreuz, Switzerland). Relative expression levels of *PINK1* and *PRKN* were calculated by 2-∆∆Ct method [47] using *RPL27* as housekeeping gene for human cells and using *Actb* as housekeeping gene for mouse tissue. Values were normalized to the relative expression level of the WT control. Used primer sequences were: Human *PINK1* (5’GGAGGAACCTGCCGAGATGTTCC-3’, 5’CCTGGAGGTGACAAAGAGCACCG-3’), human *PRKN* (5’-GCTGTGGGTTTGCCTTCT-3’, 5’-TCCACTGGTACATGGCAGC-3’), human *RPL27* (5’GATCGCCAAGAGATCAAAGATAAAA-3’, 5’CTGAAGACATCCTTATTGACGACAGT-3’), mouse *Pink1* (5’GAGTGGGACTCAGATGGCTGTCC-3’, 5’CCAGAATGGGCTGTGGACACC TC-3’), mouse *Prkn* (5’-GATTCAGAAGCAGCCAGAGG3’, 5’-GGTGCCACACTGAACTCG-3’), and mouse *Actb* (5’-AGTGTGACGTTGACATCCGTA-3’, 5’GCCAGAGCAGTAATCTCCTTC-3’).

### Data analysis

Western blot signals were quantified with ImageStudio Lite software (version 5.2.5). Data analysis was performed using GraphPad Prism (version 9.2.0). Statistical analysis for cells and rodent data with a sample size of *n* ≤ 5 was performed with parametric unpaired, student’s t-test or one-way ANOVA combined with Tukey’s test for multiple comparison or Bonferroni correction (ns, *p* > 0.05; *p < 0.05; **p < 0.005; ***p < 0.0005). The statistical analysis of rodent data with a sample size of *n* ≥ 5 was performed with a Kruskal-Wallis test followed by Dunn’s multiple comparison test (ns, *p* > 0.05; *p < 0.05; **p < 0.005; ***p < 0.0005).

## Supplementary Material

Watzlawik_SupplementaryFiles_R3_final.docx

## Abbreviations

CCCP: carbonyl cyanide m-chlorophenylhydrazone
DA: dopamine
ELISA: enzyme-linked immunosorbent assay
GAPDH: glyceraldehyde-3-phosphate dehydrogenase
GFP: green fluorescent protein
iPSCs: induced pluripotent stem cells
IQR: interquartile range
KO: knockout
MFN2: mitofusin 2
MSD: Meso Scale Discovery
p-S65-Ub: serine-65 phosphorylated ubiquitin
PD: Parkinson disease
PINK1: PTEN induced kinase 1
PRKN: parkin RBR E3 ubiquitin protein ligase
SD: standard deviation
Ub: ubiquitin
WT: wild type
