# Supplementary material for: Basal activity of PINK1 and PRKN in cell models and rodent brain
Source: Autophagy. 2023 Dec 2;20(5):1147–58. doi: 10.1080/15548627.2023.2286414 (PMC11135862; doi:10.1080/15548627.2023.2286414)
Supplement: Watzlawik_SupplementaryFiles_R3_final.docx [file KAUP_A_2286414_SM6595.docx]

# SUPPLEMENTARY MATERIAL

# Basal activity of PINK1 and PRKN in cell models and rodent brain

Jens O. Watzlawik^1,#^, Fabienne C. Fiesel^1,2,#^, Gabriella Fiorino^1,2,#^, Bernardo A. Bustillos^1^, Zahra Baninameh^1^, Briana N. Markham^1^, Xu Hou^1^, Caleb S. Hayes^1^, Jenny M. Bredenberg^1^, Nicholas W. Kurchaba^1^, Dominika Fričová^1^, Joanna Siuda^3^, Zbigniew K. Wszolek^4^, Sachiko Noda^5^, Shigeto Sato^5^, Nobutaka Hattori^5^, Asheeta A. Prasad^6^, Deniz Kirik^6,7^, Howard S. Fox^8^, Kelly L. Stauch^8^, Matthew S. Goldberg^9^ and Wolfdieter Springer^1,2,^*

Affiliations:

^1^ Department of Neuroscience, Mayo Clinic, Jacksonville, FL 32224, USA

^2^ Neuroscience PhD Program, Mayo Clinic Graduate School of Biomedical Sciences, Jacksonville, FL 32224, USA

^3^ Department of Neurology, Faculty of Medical Sciences in Katowice, Medical University of Silesia, Katowice 40-055, Poland

^4^ Department of Neurology, Mayo Clinic, Jacksonville, FL 32224, USA

^5^ Department of Neurology, Juntendo University Graduate School of Medicine, Tokyo 113-8421, Japan

^6^ Faculty of Medicine and Health, School of Medical Sciences, University of Sydney, Sydney, NSW, Australia

^7^ Department of Experimental Medical Science, Lund University, Lund 22184, Sweden

^8^ Department of Neurological Sciences, University of Nebraska Medical Center, Omaha, NE 68198, USA

^9^ Center for Neurodegeneration and Experimental Therapeutics, Department of Neurology, University of Alabama at Birmingham, Birmingham, AL 35294, USA

^#^ Contributed equally

* Corresponding author

Correspondence should be addressed to:

Wolfdieter Springer, PhD

Department of Neuroscience

Mayo Clinic

4500 San Pablo Road

Jacksonville

FL 32224, USA

E-mail: Springer.Wolfdieter@mayo.edu

Phone: +1-904-953-6129

Fax: +1-904-953-7117

**Running title:** PINK1 and PRKN are active at basal conditions.

#
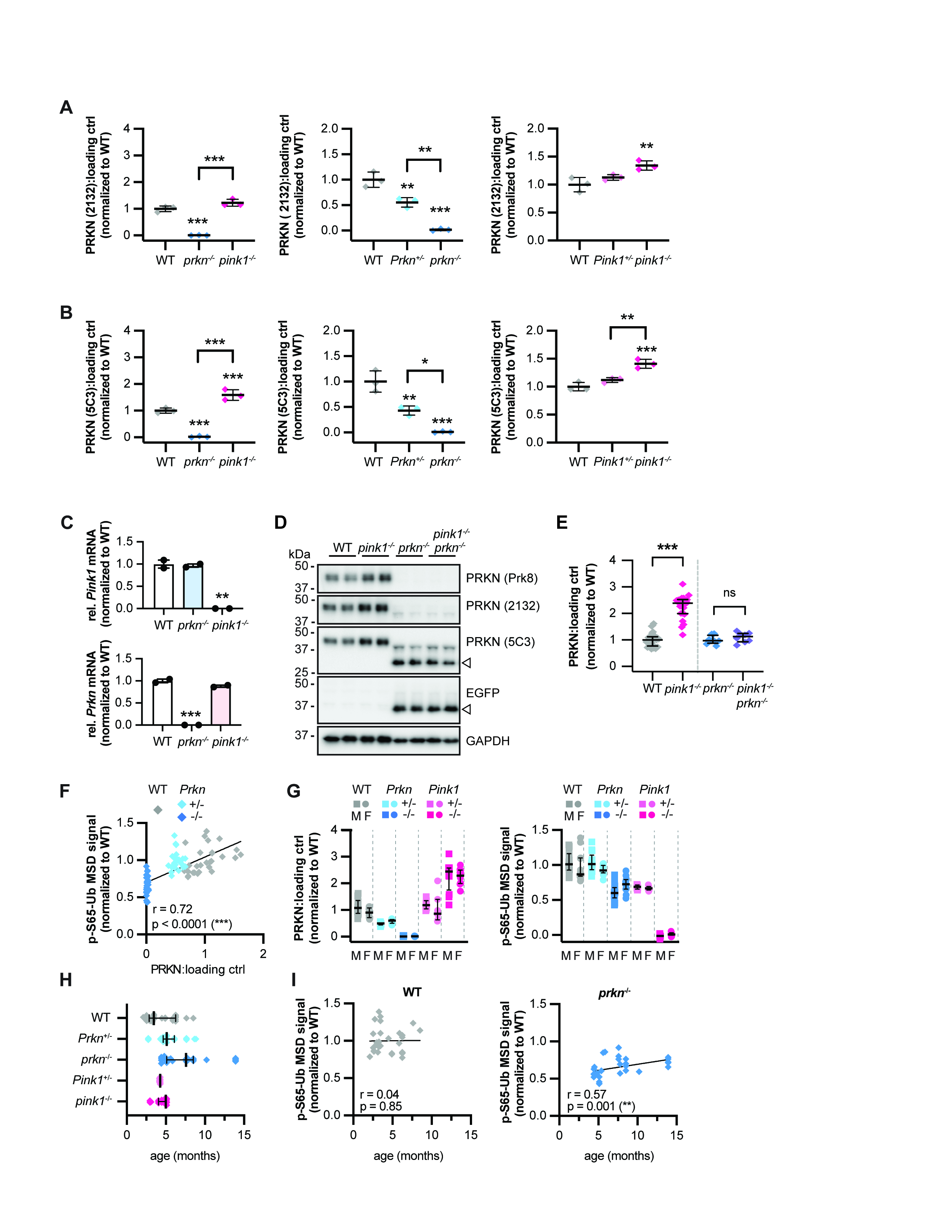
Figure S1. Basal PINK1-PRKN activity in WT and knockout mouse brain. (A and B) Quantification of PRKN protein by western blot of hemibrain lysates of the following genotype groups: WT, *prkn^-/-^*, *pink1^-/-^* (left); WT, *Prkn^+/-^*, *prkn^-/-^* (middle); and WT, *Pink1^+/-^*, *pink1^-/-^* (right) mice (n = 3 per genotype). Western blots were presented in Fig. 1A with (A) anti-PRKN (2132) and (B) anti-PRKN (5C3) showing 1.34-fold increased PRKN levels (2132; p=0.0097) and 1.41-fold increased PRKN levels (5C3; p=0.0008) respectively in *pink1^-/-^* vs. WT brain (right panels). Data points are shown as ratio of PRKN divided by GAPDH (mean ± SD). Data was analyzed by one-way ANOVA combined with Tukey’s multiple comparison test (***, p<0.0005; **, p<0.005; *, p<0.05). (C) *Pink1* and *Prkn* mRNA levels were determined by qRT-PCR from WT, *prkn^-/-^*, and *pink1^-/-^* hemibrain homogenates. Transcripts containing *Pink1* exon 6-8 or *Prkn* exon 3-4 were undetectable in the respective mutants but were similar in samples from WT and the other gene KO, respectively. Analysis was performed by one-way ANOVA followed by Tukey’s post-hoc test (***, p<0.0005; **, p<0.005). (D and E) Analysis of hemibrain protein lysates from *prkn^-/-^* animals (n = 8) or *pink1^-/-^;prkn^-/-^* double homozygous KO mice (n = 8), compared with samples from WT (n = 29) and *pink1^-/-^* (n = 22) animals. Full-length PRKN protein was significantly elevated in *pink1^-/-^* samples, but levels of the truncated PRKN-EGFP-fusion protein were unchanged in *pink1^-/-^;prkn^-/-^* double mutants. (D) Representative western blots with three different anti-PRKN antibodies (Prk8, 2132, and 5C3) and EGFP are shown with the loading control GAPDH. An open arrowhead labels the truncated EGFP fusion produced in the *prkn^-/-^* samples detected with the 5C3 antibody. (E) Quantification of the signal from anti-PRKN (5C3) is shown as a ratio of PRKN divided by GAPDH. Shown is the median ± IQR. Data was analyzed by Kruskal Wallis test followed by a Dunn’s post-hoc test (***, p<0.0005). (F) Spearman correlation for brain p-S65-Ub and PRKN (Prk8) divided by GAPDH protein levels in all WT, *Prkn^+/-^*, and *prkn^-/-^* mice. (G) Comparison of PRKN (Prk8) divided by GAPDH protein (left) and p-S65-Ub levels (right) in all mouse hemibrain lysates for each genotype by sex: M – males (rectangles); F – females (circles). WT levels (combined sex) were normalized to 1. Mann-Whitney test was used for pairwise analysis (***, p<0.0005; **, p<0.005; *, p<0.05). (H) Age distribution of all mice for each genotype showed that the *prkn*^-/-^ animals were significantly older than WT mice. Data is shown as the median ± IQR. Analysis was performed by Kruskal-Wallis followed by a Dunn’s post-hoc test (***, p<0.0005). (I) Spearman correlation of p-S65-Ub levels with age for WT (left) and *prkn^-/-^* (right) mice.

#
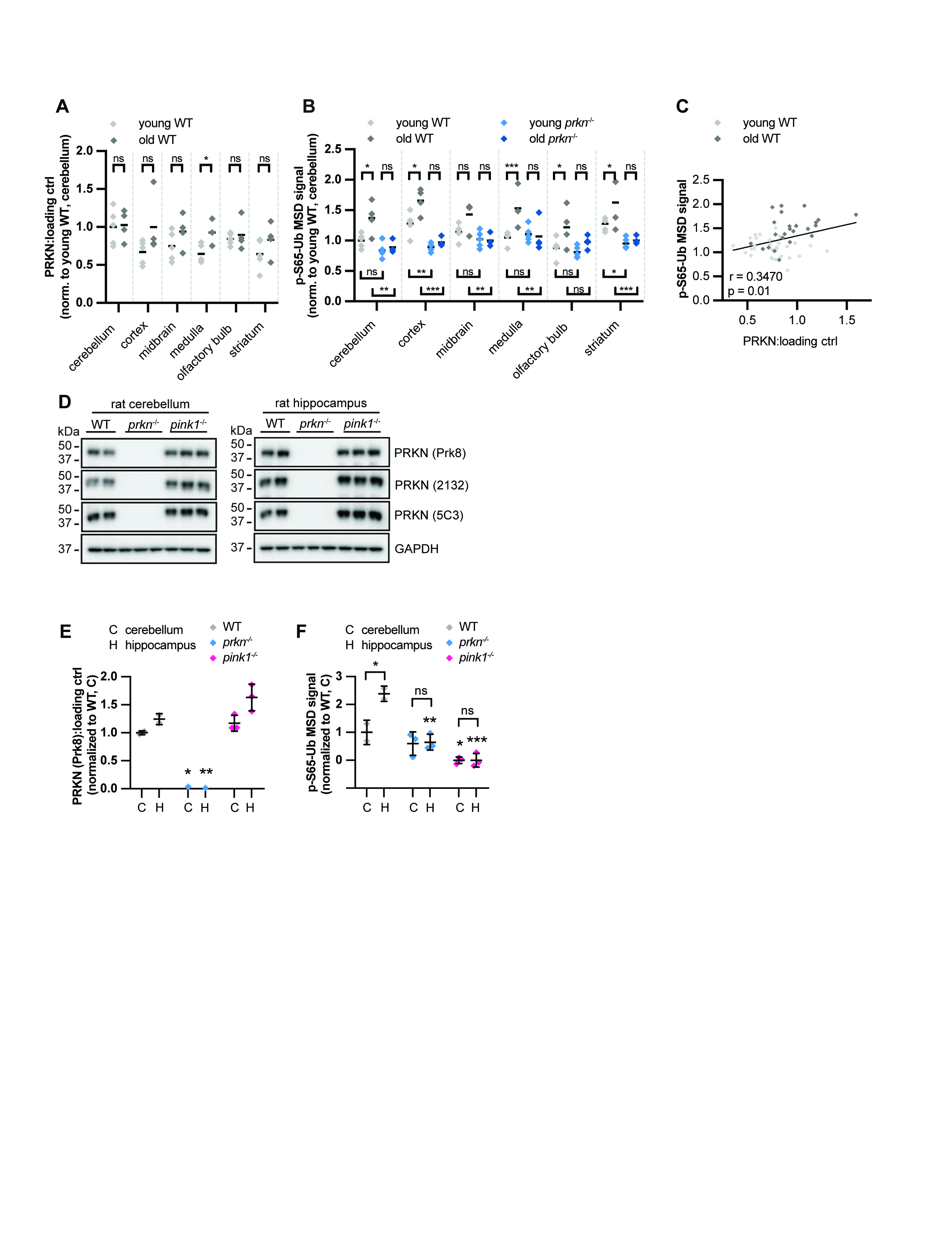
Figure S2. Regional changes of PRKN and p-S65-Ub levels in rodent brain during aging. (A-C) Brains from an independent cohort of young (4.5 months) and old (24 months) mice were dissected into six distinct subregions: cerebellum, cortex, medulla oblongata, midbrain, olfactory bulb, and striatum. (A) PRKN divided by GAPDH protein levels were determined by western blot in brain regions of young (n = 5; light gray) and old (n = 4; dark gray) WT mice. Data shown are normalized to cerebellum and were analyzed by unpaired t-test (*, p<0.05). (B) p-S65-Ub levels were determined by MSD ELISA in brain samples of the WT mice as above as well as from young (n = 5; light blue) and old (n = 4; dark blue) *prkn^-/-^* mice. Data shown are normalized to cerebellum. Data were analyzed with two-way ANOVA followed by Tukey’s test for multiple comparison (***, p<0.0005; **, p<0.005; *, p<0.05). Brackets on top of the data compare the two age groups within each genotype. Brackets at the bottom compare data across both genotypes, for each age group individually. (C) Spearman correlation of PRKN divided by GAPDH and p-S65-Ub levels in young (light gray) and old WT (dark gray) mice across all six brain subregions analyzed. (D) Shown are PRKN (Prk8, 5C3, and 2132) western blots of cerebellar (left) and hippocampal (right) brain lysates from 8 months old Long-Evans rats with homozygous loss of *Prkn* (n = 3) or *Pink1* (n = 3) compared to WT (n = 2). (E) PRKN (Prk8) protein levels were determined by densitometric analysis and the data is shown as a ratio of PRKN divided by GAPDH in both brain regions. (F) Graph shows p-S65-Ub levels measured by MSD ELISA in rat cerebellar and hippocampal lysates. (E and F) Shown is the mean ± SD. Statistical analysis was performed with one-way ANOVA followed by Tukey’s post-hoc test. Asterisks on top of the data points indicate significant difference to WT, asterisks with brackets indicate comparison between cerebellum and hippocampus within the same genotype (***, p<0.0005; **, p<0.005; *, p<0.05; ns, not significant).


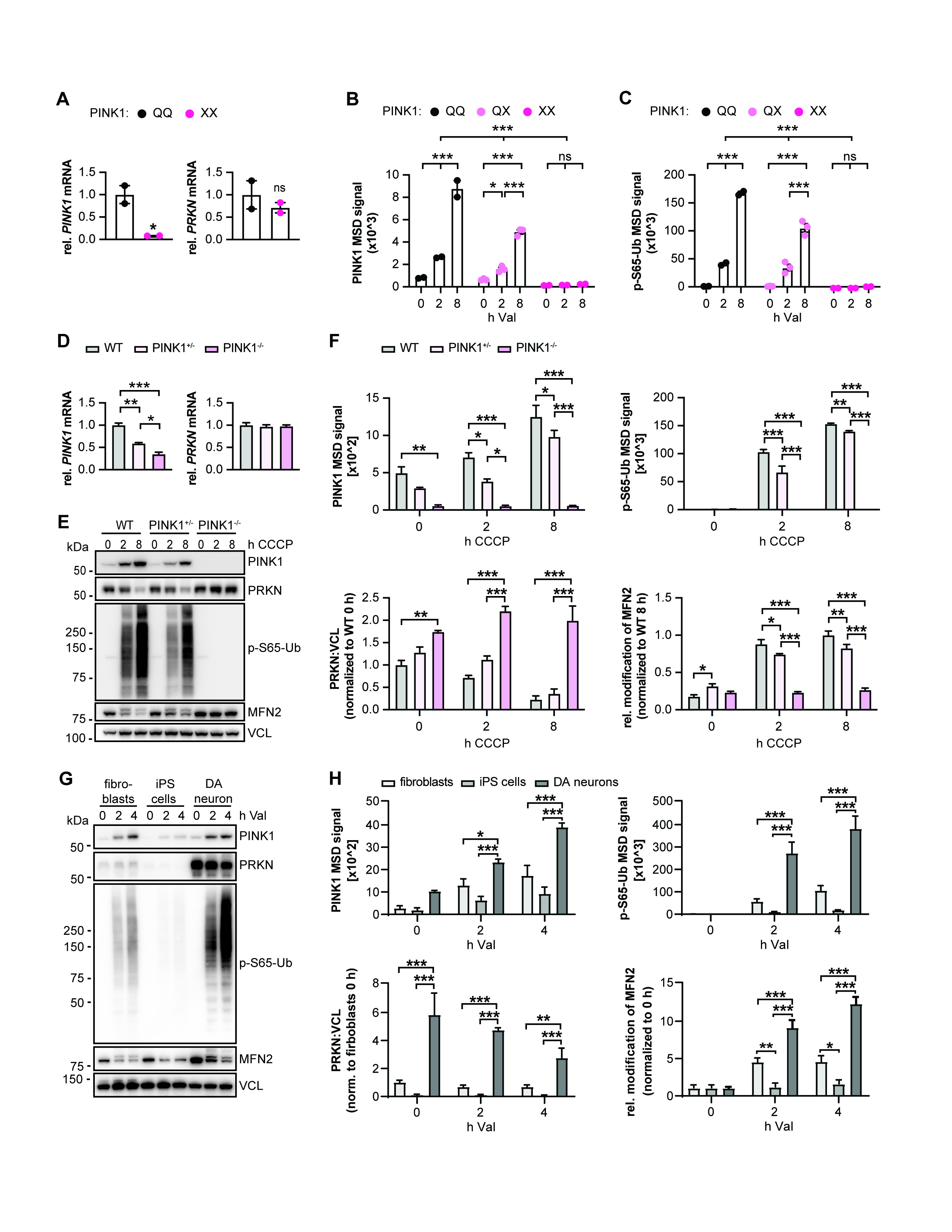
**Figure S3.** PINK1-PRKN activity in human fibroblasts, iPSCs and dopaminergic neurons. (**A**) *PINK1* (left) and *PRKN* (right) mRNA levels were measured from human fibroblasts without PINK1 mutation (QQ) and from homozygous PINK1 mutant cells (XX) and are each shown as fold change compared to WT. Statistical analysis was performed by unpaired t-test (*, p<0.05). (**B**) PINK1 protein and (**C**) the respective p-S65-Ub levels were measured by ELISA from fibroblasts with zero (WT; QQ), one (QX) or two (XX) mutant PINK1^Q456X^ alleles. This data was presented per time point in Fig. 2B and 2D, respectively, and is arranged here by genotype group over the entire treatment course with 1 µM valinomycin (Val). (**B and C**) Statistical analysis was performed by two-way ANOVA followed by Tukey’s post-hoc test. Over the entire time course, all three genotypes are significantly different from each other (***, p<0.0005; *, p<0.05). (**D**) mRNA levels were measured from ReN cell VM differentiated into neurons with hetero- (+/-) or homozygous (-/-) loss of PINK1 and are each shown as fold change compared to WT. Graphed is the mean ± SD of three independent experiments. Statistical analysis was performed by one-way ANOVA followed by Tukey’s post-hoc test (***, p<0.0005; **, p<0.005; p<0.05). (**E and F**) Differentiated neurons with loss of only one (+/-) or both (-/-) PINK1 alleles were analyzed at basal conditions and after a 2- or 8-hour treatment with 20 µM CCCP. Representative immunoblots document the respective protein levels of PINK1, PRKN, p-S65-Ub, and the PRKN substrate MFN2. (**F**) Quantification by protein levels by MSD (PINK1 and p-S65-Ub) or densitometry of western blots (PRKN and MFN2). PRKN protein levels are shown as a ratio of PRKN divided by VCL. The relative modification of MFN2 was calculated by dividing the density of the upper (ubiquitinated) band by the lower (unmodified) band. Shown is the mean ± SD of three independent experiments. (**G**) Representative immunoblots document the respective protein levels of PINK1, PRKN, p-S65-Ub, and MFN2 in fibroblasts, iPSCs and DA neurons over the time course with 1 µM Val treatment. (**H**) Protein levels of the different cell types were quantified by MSD (PINK1 and p-S65-Ub) or densitometry of western blots (PRKN and MFN2) at basal conditions and after 2- or 4-h Val treatment. PRKN protein levels are shown as a ratio of PRKN divided by VCL and further adjusted to the relative expression level of a WT iPSC calibrator included in each experiment. The relative modification of MFN2 was calculated as above. Shown is the mean ± SD of three independent experiments per cell type. Statistical analysis for (**F**) and (**H**) was performed by two-way ANOVA followed by Tukey’s test for multiple comparisons (***, p<0.0005; **, p<0.005; *, p<0.05).
